# MicroRNA-126 regulates the induction and function of CD4^+^ Foxp3^+^ regulatory T cells through PI3K/AKT pathway

**DOI:** 10.1111/jcmm.12003

**Published:** 2013-01-10

**Authors:** Andong Qin, Zhenke Wen, Ya Zhou, Ying Li, Yongju Li, Junmin Luo, Tao Ren, Lin Xu

**Affiliations:** a Department of Immunology, Zunyi Medical CollegeGuizhou, 563000, China; b Institute for Immunobiology and Department of Immunology, Shanghai Medical College of Fudan UniversityShanghai, 200032, China; c Department of Medical Physics, Zunyi Medical CollegeGuizhou, 563000, China; d Department of Respiratory Medicine, East Hospital, Tongji University School of MedicineShanghai, 200120, China

**Keywords:** MiR-126, CD4^+^ regulatory T cell, AKT, Adoptive cell transfer

## Abstract

Recent evidence showed that limited activation of PI3K/Akt pathway was critical for induction and function sustainment of CD4^+^Foxp3^+^ regulatory T cells (Tregs). However, the underlying mechanism remains largely unknown. In this study, we reported that miR-126 was expressed in mouse and human Tregs. Further study showed that silencing of miR-126 using miR-126 antisense oligonucleotides (ASO) could significantly reduce the induction of Tregs *in vitro*. Furthermore, miR-126 silencing could obviously reduce the expression of Foxp3 on Tregs, which was accompanied by decreased expression of CTLA-4 and GITR, as well as IL-10 and TGF-β, and impair its suppressive function. Mechanistic evidence showed that silencing of miR-126 enhanced the expression of its target p85β and subsequently altered the activation of PI3K/Akt pathway, which was ultimately responsible for reduced induction and suppressive function of Tregs. Finally, we further revealed that miR-126 silencing could impair the suppressive function of Tregs *in vivo* and endow effectively antitumour effect of CD8^+^T cells in adoptive cell transfer assay using a murine breast cancer model. Therefore, our study showed that miR-126 could act as fine-tuner in regulation of PI3K-Akt pathway transduction in the induction and sustained suppressive function of Tregs and provided a novel insight into the development of therapeutic strategies for promoting T-cell immunity by regulating Tregs through targeting specific miRNAs.

## Introduction

CD4^+^CD25^+^ regulatory T cells (Tregs), a subpopulation of CD4^+^ T cells which constitutively expressed transcription factor forkhead box protein3 (Foxp3), consist of 5–10% peripheral CD4^+^T cells in normal human and mice. A large body of literatures showed that Tregs played a critical role in various immune diseases through regulating the function of conventional CD4^+^T cells and CD8^+^ T cells [1, 2]. Foxp3 plays a critical role in development and function of Treg cells [3, 4]. Furthermore, ectopic expression of Foxp3 is sufficient, in certain experimental settings, to impart suppressive function to conventional CD4^+^T cells [5, 6]. The intrinsic mechanisms is described that Foxp3 coordinates the Treg cell gene expression program [7, 8].

Recent evidence showed that limited PI3K-Akt pathway played a critical role in the expression of Foxp3 and subsequently regulated the induction and suppressive function of Tregs [9, 10]. Efficient Foxp3 induction requires proteins that limit PI3K activity [11]. Consistently, constitutive Akt activity not only interfered with Foxp3 induction in CD4^+^T cells but also impaired the suppressive function of Tregs [12, 13]. However, the exact reason why limited activation of PI3K-Akt pathway was in the induction and sustained suppressive function of Tregs is currently unclear.

MiR-126 is one of the miRNA most abundantly expressed in endothelial cells, and heart, lung and other highly vascularized murine tissue [14–16]. Overexpression of miR-126 could block PI3K-Akt activation, and trigger ischaemic angiogenesis, suppress tumour growth [17–19]. Recent evidence further showed that miR-126 was also functional expressed in T cells [20]. And silencing of miR-126 could abrogate the function of CD4^+^Th2 cells [21]. However, the possible functional role of miR-126 in Tregs remains to be elucidated. In this study, we reported that miR-126 was expressed on murine and human Tregs. Importantly, silencing of miR-126 could reduce the induction of Foxp3 in CD4^+^T cells and impair suppressive function of Tregs. Further evidence showed that miR-126 regulated the transduction of PI3K-Akt pathway, which ultimately influenced the induction and sustained suppressive function of Tregs. Finally, we reported that miR-126 silencing in Tregs could endow the effective antitumour effect of CD8^+^ T cells in adoptive cell transfer assay. These data demonstrated a previously unknown role of miR-126 in Tregs induction and function sustainment through regulating the transduction of PI3K-Akt pathway and provided a novel insight into the development of therapeutic strategy for promoting T-cell immunity by regulating Tregs through targeting specific miRNAs.

## Material and methods

### Animals

Female Balb/c mice and Balb/c nude mice 5–6 weeks of age were purchased from the Center of Experimental Animal, Fudan University (Shanghai, China). All animals were housed in the pathogen-free mouse colony at our institution and all animal experiments were performed according to the guidelines for the Care and Use of Laboratory Animals (Ministry of Health, PR China, 1998) and all the experimental procedure was approved by the ethical guidelines of Shanghai Medical Laboratory Animal Care and Use Committee.

### Cell lines and reagents

4T1 cell line, a Balb/c spontaneous metastatic mammary carcinoma, was cultured at 37°C under 5% CO_2_ in completed RPMI 1640 (GIBICO, Grand land, NY, USA) medium containing 10% heat-inactivated foetal bovine serum and supplemented with 2 mM glutamine, 100 IU/ml penicillin and 100 μg/ml streptomycin sulfate. 2′-OMe-modified antisense oligonucleotides of miR-126 (termed as miR-126 ASO: GCAUUAUUACUCACGGUACGA) and corresponding scramble control (Integrated DNA Technologies, Commercial Park Coralville, IA, USA), miR-126 mimics and corresponding scramble controls were purchased from Ambion. p85β RNAi and corresponding scramble controls (Santa Cruz biotechnology. Inc, Delaware Avenue, CA, USA), Akt inhibitor IV was purchased from Merck. All other reagents were purchased from Sigma-Aldrich (St. Louis, MO, USA) unless stated otherwise.

### PBMCs

Peripheral blood was obtained from healthy donor (*n* = 18) at the age of 20–25. Blood was drawn into heparin containing vacutainer tubes (Becton Dickinson, Franklin Lakes, NJ, USA), diluted 2:1 with Dulbecco's phosphate-buffered salt solution 1× without calcium or magnesium (Mediatech, Oceanside, CA, USA) and then separated by centrifugation over a ficoll (Pharmacia Biotech AB, Centennial Ave. Piscataway, NJ, USA) density gradient for 20 min. at 1000 *g* at room temperature. PBMCs were collected, washed and resuspended in RPMI 1640 (containing 10% FCS) for future use.

### Nude mice tumour model and cotransfer experiment

This experiment was performed as previously described with a little modification [22]. Syngeneic female nude mice were injected subcutaneously with 0.2 ml of a single-cell suspension containing 2 × 10^5^ 4T1 adenocarcinoma cells in the right anterior mammary fat pad region. Seven days later, 4T1 specific CD8^+^T cells were transferred with or without Tregs at a ratio of 2:1 through tail vein respectively. Twenty-one days later, the size of tumour mass in each group was obtained. To obtain 4T1 specific CD8^+^T cells, Balb/c mice were immunized s.c by 1 × 10^6^ mitomycin C-treated 4T1 cells three times with 7 days interval. Ten days later, splenocytes were harvested and restimulated by mitomycin C-treated 4T1 cells for 24 h in the presence of IL-2 (50 U/ml) *in vitro*. Then, 2 × 10^6^ 4T1 specific CD8^+^ T cells were purified by FACS sorting and adoptively transferred into 4T1 bearing syngeneic nude mice.

### Flow cytometry

Flow cytometry was performed on a FACSAria (BD Biosciences, Qume Drive, CA, USA) with CellQuest Pro software (BD Biosciences) using directly conjugated mAbs against the following human or murine markers: CD4-PerCP, CD25-allophycocyanin, CD127-PerCP-Cy5.5, CTLA-4-FITC and GITR-FITC with corresponding isotype-matched controls (either BD Biosciences or eBioscience Systems, Science Center Drive, CA, USA). Foxp3 staining was conducted using the Human or Murine Regulatory T cell staining kit (eBioscience) and run according to the manufacturer's protocol.

### CD4^+^ Th cell differentiation

For iTreg differentiation, naive CD4^+^CD25^−^ T cells purified by negative selection (Miltenyi Biotec, Robert-Koch-Str, Teterow, Germany) were stimulated by plate-bound anti-CD3 (2 μg/ml) and anti-CD28 mAbs (4 μg/ml) in the presence of 5 ng/ml TGF-β and 2 ng/ml IL-2 (BD Biosciences). After 5 days, cells were stained for CD4 and Foxp3. For Th0 differentiation, CD4^+^CD25^−^ T cells were stimulated by plate-bound anti-CD3 (2 μg/ml) and anti-CD28 mAbs (4 μg/ml).

### Real-time PCR

RNA was extracted from purified cells with Trizol (Invitrogen, Col Anzures, Mexico, CA, USA) according to manufacturer's recommendations. cDNA was prepared with Superscipt II reverse transcriptase (Invitrogen) using an oligo dT primer. The following specific primers were used: murine HPRT(fwd): 5′-TGACACTGGCAAAACAATGCA-3′, murine HPRT(rev): 5′-GGTCCTTTTCACCAGCAAGCT-3′; murine IL10(fwd):5′-TGGCATGAGGATCAGCAGGG-3′, murine IL10(rev): 5′-GGCAGTCCGCAGCTCTAGG-3′; murine TGF-β (fwd): 5′-GCGTGCTAATGGTGGA-3′, murine TGF-β (rev): 5′-CAGGCGTATCAGTGGG-3′; Human HPRT(fwd): 5′-ACATTGTAG- -CCCTCTG-3′, Human HPRT(rev): 5′-GGATTATACTGCCTGA-3′; Human IL10(fwd):5′-GA- -GAACCAAGACCCAGAC-3′, Human IL10(rev):5′-CACAGGGAAGAAATCG-3′; Human TGF-β (fwd): 5′-CCCACAACGAAATCTATGA-3′, Human TGF-β (rev): 5′-GAGGTAT- -CGCCAGGAAT-3′. The reaction mixture consisted of 2 μl of LightCycler™ DNA Master SYBR Green I (Roche Diagnostic Company, Indianapolis, IN, USA). The cycling program had a 10 min. initial denaturation at 95°C and then entered a cycle of an instant 95°C denaturation, 10 sec. of annealing at 60°C and 12 sec. of extension at 72°C with transition rate of 20°C/sec. between temperature plateaus for a total of 40 cycles. Quantification of data was analysed using the LightCycler analysis software version 4.5.

### Transient transfection

miR-126 ASO, p85β RNAi and the corresponding control oligonucleotides were purchased. For transient transfection, 10 ng (or 5 ng) of synthesized oligonucleotide was mixed with 100 μl of T-cell Nucleofector solution (Amaxa, Gaithersburg, MD, USA) and transfected into 2 × 10^6^ cells by electroporation using a Nucleofector II instrument. The transfection efficiency was around 65%, evaluated by flow cytometric analysis after a fluorescence-labelled ASO control or siRNA control (Dharmacon, Lafayette, CO, USA). After transfection, the cells were allowed to recover by incubating for 4 h at 37°C, and then used for the following assays.

### Retroviral transduction

293FT cells (Invitrogen) were transfected with retroviral expression plasmids (MSCV IRES-GFP), either empty or encoding the miR-126 ASO and the packaging construct pcl-ECO using TransIT-293 (Mirus, Temple Avenue, London, UK) as previously described [13], according to the manufacturer's instructions. Naive CD4^+^ CD25^−^ T cells were activated in the presence of anti-CD3 (2 μg/ml) and anti-CD28 mAbs (4 μg/ml) in the presence of 5 ng/ml TGF-β and 2 ng/ml IL-2 and infected with retrovirus supernatant at 24 hrs. Cells were subsequently cultured for an additional 96 hrs for a total *in vitro* culture of 5 days.

### Suppression assays

To test Tregs suppressive activity, 5 × 10^4^ CD4^+^CD25^−^ cells were treated with 2 μg/ml anti-CD3 (eBioscience) and anti-CD28 (eBioscience) for 12 hrs as effector cells, then incubated with or without Tregs at a ratios of 2:1 for 72 hrs in complete medium containing RPMI 1640 (Sigma-Aldrich, St. Louis, MO, USA) supplemented with 5% FCS. [^3^H] thymidine (0.5 μCi/well) was added for the last 18 hrs of culture.

### Western blotting

Equal amounts of protein were resolved under reducing conditions on a 12% SDS–polyacrylamide gel. Protein migration was assessed using protein standards (Biorad, Hercules, CA, USA). Transfer to a nitrocellulose membrane was performed overnight at 30 V using a wet transfer system. Equal protein loading was confirmed with Ponceau staining. The membrane was washed in 5% skim milk in phosphate-buffered saline+0.03% Tween 20 (PBS-T) for 1 hr to block non-specific protein binding sites on the membrane. Immunoblotting was performed using a monoclonal antibody to p85β (ab62901; Abcam, Cambridge Science Park, Cambridge, UK), Akt (SC-8312; Santa Cruz Biotechnology), phosphorylation of Akt (pAkt, Ser 473) (SC-33437; Santa Cruz Biotechnology), mTOR (ab2732; Abcam), phosphorylation of mTOR (pmTOR, ab45996; Abcam), total p85 (42925; Cell Signaling Technology, Danvers, MA, USA) and p85α (ab22653; Abcam) at a dilution of 1/1000 in a non-fat milk-Tris buffer. The membrane was then washed in PBS-T and subsequently probed with a secondary anti-rabbit or antimouse antibody conjugated to horseradish peroxidase (Amersham Life Sciences, Piscataway, NJ, USA) at a dilution of 1:5000 and developed with chemiluminescence (Pierce, Rockford, IL, USA). The membrane was then exposed to X-ray film (Kodak, Rochester, NY, USA) which was subsequently developed.

### BrdU labelling

The 4T1 bearing syngeneic nude mice were treated with 2 mg BrdU (5-bromo-2-deoxyuridine; Sigma-Aldrich) i.p. every other day up to a cumulative dose of 8 mg BrdU as indicated. Eight hours after the last BrdU injection, TILs recovered from the tumour mass were analysed by flow cytometry for their incorporation of BrdU. In brief, TILs were stained with antibodies for cell surface markers and fixed and permeabilized using Cytofix/Cytoperm and Perm/Wash buffer (BD Biosciences) according to the manufacturer's instructions. Cells were incubated at 24°C for 30 min. in 0.15 M NaCl, 4.2 mM MgCl_2_, 10 mM HCl, pH5 in the presence of 2 U DNase I (Invitrogen), followed by staining with αBrdU-FITC (eBioscience) for 30 min. and were finally analysed by FACS.

### Intracellular staining for IFN-γ and Granzyme B

TILs were isolated from 4T1 bearing syngeneic nude mice transferred with CD8^+^T cells or CD8^+^T cells plus Tregs, respectively, at the indicated time-point. After staining of surface markers (CD8), cells were fixed and permeabilized using Cytofix/Cytoperm and Perm/Wash buffer from BD Biosciences according to the manufacturer's instructions. Antibodies to IFN-γ or Granzyme B and corresponding isotype controls were obtained from BD Biosciences. Cells were stained with antibody against IFN-γ (1:100) or antibody against Granzyme B (1:100) at 25°C for 20 min. and washed twice in Perm/Wash before analysis.

### CFSE staining

Cells were labelled with 5-carboxy-fluorescein diacetate succinimidyl ester (CFSE; Molecular Probes, Leiden, The Netherlands) as previously described [22]. A total of 2 × 10^5^ CFSE-labelled cells were cultured in 24-well plate in the condition of anti-CD3/CD28. Ninety-six hours later, the proliferation of cells was analysed by FACS.

### Statistical analyses

Statistical analyses of the data were performed with the aid of analysis programs in SPSS12.0 software. Statistical evaluation was performed using one-way analysis of variance (anova) or *t*-test using the program PRISM 4.0 (GraphPad Software Inc., San Diego, CA, USA). The *P* values <0.05 were considered significant and are indicated on the figures accompanying this article as follows unless otherwise indicated: *P < 0.05. Unless otherwise indicated, error bars represent SD.

## Results

### The expression of miR-126 on Tregs

**Fig. 1:**
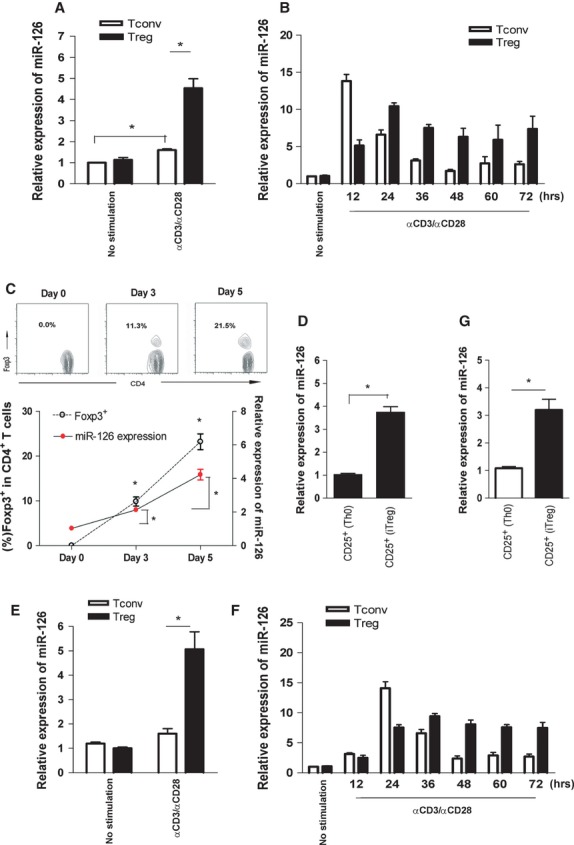
The expression of miR-126 on Tregs. (**A**) CD4^+^ CD25^−^ T cells (Tconv) and CD4^+^ CD25^+^ T cells (Tregs) were purified by MACS sorting from splenocytes of normal Balb/c mice and cultured in the presence of anti-CD3/CD28 for 48 h. The expression of miR-126 was detected by Real-time PCR assay. (**B**) CD4^+^ CD25^−^ T cells (Tconv) and CD4^+^CD25^+^ T cells (Tregs) were cultured in the presence of anti-CD3/CD28. The expression of miR-126 was detected by Real-time PCR assay at indicated time-point. (**C**) CD4^+^ CD25^−^ T cells were isolated from splenocytes of normal Balb/c mice and stimulated with anti-CD3/CD28 antibody in the presence of TGF-β and IL-2. The proportion of Foxp3^+^ cells in CD4^+^ T cells and the expression of miR-126 were analysed at indicated time-point. (**D**) CD4^+^ CD25^−^ T cells were isolated from splenocytes of normal Balb/c mice and stimulated with anti-CD3/CD28 antibody for Th0 differentiation and TGF-β plus IL-2 for Tregs differentiation Five days later, CD25^+^ cells in Th0 differentiation condition or in iTregs differentiation condition were isolated by MACS respectively and the expression of miR-126 was determined by Real-time PCR assay. (**E** and **F**) CD4^+^ CD25^−^ T cells (Tconv) and CD4^+^ CD25^high^ CD127^−^ T cells (Tregs) were purified from PBMCs of health donors (*n* = 6) by FACS sorting and cultured in the presence of anti-CD3/CD28. The expression of miR-126 was detected using Real-time PCR at 48 h or indicated time-point. (**G**) CD4^+^ CD25^−^ T cells were purified from PBMCs from healthy donors (*n* = 6) by FACS sorting and cultured in Th0 or Treg differentiation condition. After 5 days, CD25^+^ cells in each differentiation condition were isolated by MACS, respectively, and the expression of miR-126 was determined by Real-time PCR assay. One representative of three independent experiments was shown. *P < 0.05.

To investigate the possible role of miR-126 on CD4^+^Foxp3^+^ regulatory T cells (Tregs), we first detected the expression level of miR-126 in Tregs by Real-time PCR assay. Our data showed that fresh Tregs and fresh Tconv expressed similar level of miR-126 (Fig. 1A). After 48 hrs of activation, the expression level of miR-126 in Tregs was higher than that in Tconv (Fig. 1A, *P* < 0.05). To analyse the kinetics of miR-126 expression upon activation, a time course experiment was performed. The expression of miR-126 on both Tregs and Tconv also were up-regulated upon 12 hrs of activation (Fig. 1B, *P* < 0.05). In Tconv cells, the miR-126 level peaked after 12 hrs and rapidly decreased after 24 hrs, then reached a plateau even after 72 hrs of activation. In Tregs, the maximal expression level was reached after 36 hrs of activation and remained at this moderate plateau for the duration of the experiment. Next, we further detected the expression of miR-126 on inducible Tregs (iTregs). As shown in Figure 1C, the expression of miR-126 increased during the induction of Tregs, accompanied by elevated proportion of CD4^+^Foxp3^+^ T cells *in vitro* experimental system wherein the activation of CD4^+^ CD25^−^ cells in the presence of TGF-β and IL-2 induces *de novo* expression of Foxp3 (*P* < 0.05). On day five, we further isolated CD25^+^ T cells in iTregs differentiation condition, which highly expressed Foxp3 and could potently inhibit the proliferation of CD4^+^CD25^−^T cells (data not shown), and found that CD25^+^ T cells also expressed higher level of miR-126 compared with CD25^+^ T cells in Th0 differentiation condition (Fig. 1D, *P* < 0.05), indicating miR-126 also was highly expressed on iTregs. To confirm this finding in human, CD4^+^CD25^high^ CD127^−^Tregs were isolated from PBMCs. Consistently, similar results also were obtained on Tregs and Tconv before or after activation (Fig. 1E and F). Finally, human CD25^+^ T cells (iTregs) also expressed high level of miR-126 (Fig. 1G, *P* < 0.05). All these data suggested that Tregs expressed miR-126 which might be involved in the induction and function of Tregs.

### Silencing of miR-126 reduced the induction of Tregs

**Fig. 2:**
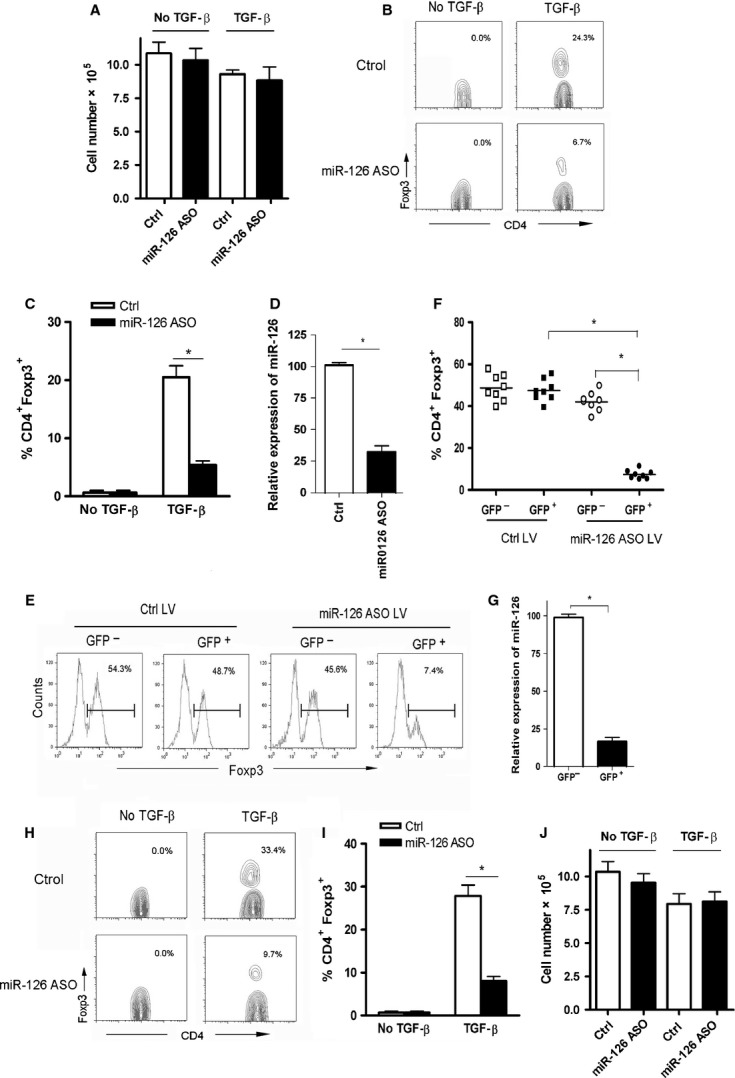
Silencing of miR-126 reduced the induction of Tregs. CD4^+^ CD25^−^ T cells were isolated from splenocytes of normal Balb/c mice and stimulated with anti-CD3/CD28 antibody in the presence of TGF-β and IL-2 for 24 h, and then were transiently transfected with miR-126 ASO (10 ng) or scramble control (10 ng). After 4 days, the total cell numbers were calculated (**A**) and the proportion of Foxp3^+^ cells in CD4^+^ T cells was analysed by FACS (**B**) and calculated (**C**). (**D**) The expression of miR-126 in miR-126 ASO-transfected or control group was detected by Real-time PCR assay. CD4^+^ CD25^−^ T cells infected with retrovirus encoding either empty vector control (Ctrl-LV) or miR-126 ASO (miR-126 ASO LV) were stimulated with anti-CD3/CD28 antibody in the presence of TGF-β and IL-2. After 4 days, the expression level of Foxp3 was analysed by FACS (**E**) and the percentages of cells positive for Foxp3 are shown (**F**). (**G**) The expression of miR-126 in miR-126 ASO LV infected or Ctrl-LV infected Tregs was detected by Real-time PCR assay. CD4^+^CD25^−^ T cells were purified from PBMCs from healthy donors (*n* = 6) by FACS sorting and cultured in the condition of induction of Tregs for 24 h and then were transiently transfected with miR-126 ASO (10 ng) or scramble control (10 ng). Four days later, the proportion of Foxp3^+^ cells in CD4^+^ T cells was analysed by FACS (**H**) and calculated (**I**). And the total cell numbers also were calculated (**J**). One representative of three independent experiments was shown. *P < 0.05.

We then investigated the possible role of miR-126 in the induction of Tregs using miR-126 antisense oligonucleotides (ASO). As shown in Figure 2A, the cell number in miR-126 ASO-transfected group was equal to those in control groups in the end of culture (*P* > 0.05), suggesting that miR-126 ASO did not influence the proliferation of cells. Notably, we found that the proportion of Foxp3^+^ in CD4^+^ T cells decreased significantly in miR-126 ASO-transfected group compared with that in control group (Fig. 2B and C, *P* < 0.05). To identify the inhibition efficiency of miR-126 ASO, we also detected and found the expression of miR-126 in miR-126 ASO-transfected group decreased about 70% (Fig. 2D, *P* < 0.05). Then, to further confirm these findings, we constructed retrovirus expression miR-126 ASO (termed as miR-126 ASO LV) and encoded a GFP marker which allowed the discrimination between infected and non-infected cells in the same cultures. As shown in Figure 2E and F, the effect of miR-126 ASO was specific and intrinsic for induction of Tregs, which was demonstrated by the impaired induction in miR-126 ASO-expressing cells (GFP^+^ infected cells), but not GFP^−^ uninfected cells. Furthermore, the observed impairment was also not caused by a perturbation of the overall activation of the cells as similar cell numbers were observed at the end of the culture (data not shown). Moreover, we also found that the expression of miR-126 in miR-126 ASO-expressing cells (GFP^+^ infected cells) decreased significantly compared with GFP^−^ uninfected cells (Fig. 2G, *P* < 0.05).

We performed similar experiments in human CD4^+^CD25^−^T cells. As shown in Figure 2H and I, the proportion of Foxp3^+^ in CD4^+^ T cells decreased obviously in miR-126 ASO-transfected group compared with that in control group (9.42 ± 0.87 *versus* 27.83 ± 2.11, *P* < 0.05). In line with our above findings, miR-126 ASO transfection significantly reduced the expression of miR-126 (data not shown), but did not alter the total cell number in each group (Fig. 2J, *P* > 0.05). Combining these data strongly suggested that silencing of miR-126 could significantly reduce the induction of Tregs.

### Silencing of miR-126 altered the expression of Foxp3 in Tregs

**Fig. 3:**
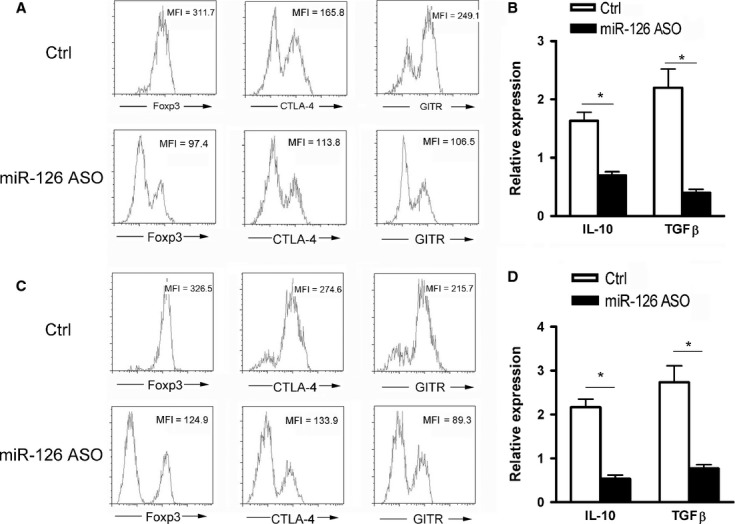
Silencing of miR-126 altered the expression of Foxp3 and functional molecules on Tregs. CD4^+^ CD25^+^ T cells (Tregs) purified from splenocytes of normal Balb/c mice by MACS were transiently transfected with miR-126 ASO (10 ng) or Scramble control (10 ng), and then cultured in the presence of anti-CD3/CD28 plus IL-2 (50 IU/ml) for 48 h. The expression of Foxp3, GITR and CTLA-4 were analysed by FACS (**A**). The expression of IL-10 and TGF-β was determined by Real-time PCR (**B**). CD4^+^ CD25^high^ CD127^−^ T cells (Tregs) purified from PBMCs of healthy donors (*n* = 6) by FACS sorting were transiently transfected with miR-126 ASO (10 ng) or Scramble control (10 ng). Then, cells were cultured in the presence of anti-CD3/CD28 plus IL-2 (50 IU/ml) for 48 h. The expression of Foxp3, GITR and CTLA-4 were analysed by FACS (**C**). The expression of IL-10 and TGF-β also was determined by Real-time PCR (**D**). One representative of three independent experiments was shown. *P < 0.05.

Given that miR-126 silencing significantly reduced the induction of Tregs, we next sought to investigate the possible effect of miR-126 silencing on the expression of Foxp3 in Tregs. As shown in Figure 3A, the expression level of Foxp3 on Tregs in miR-126 ASO-transfected group decreased obviously compared with that in the control group, accompanied by about 70% decrease in miR-126 expression (data not shown). To confirm this finding, we also detected the expression of Foxp3 on Tregs in miR-126 ASO LV infected group. Our data showed that the expression of Foxp3 on GFP^+^Tregs was lower than that in GFP^−^Tregs (Supporting Information Figure S1, *P* < 0.05). These data suggested that miR-126 silencing could reduce the expression of Foxp3 in Tregs. Next, we further investigate the expression of function-associated molecules on Tregs in miR-126 ASO-transfected group. Our data showed that the expression of Cytotoxic T lymphocyte–associated antigen 4 (CTLA-4) and glucocorticoid-Induced Tumour Necrosis Factor Receptor (GITR), as well as IL-10 and TGF-β, on Tregs also decreased significantly (Fig. 3A and B, *P* < 0.05). We further performed similar experiment on human Tregs. As shown in Figure 3C, the expression level of Foxp3 on Tregs also decreased significantly in miR-126 ASO-transfected group, accompanied by reduced expression level of CTLA-4 and GITR, as well as IL-10 and TGF-β (Fig. 3C and D, *P* < 0.05). These data demonstrated that miR-126 silencing could impair the expression of Foxp3 in Tregs and subsequently alter its functional molecules expression.

### Silencing of miR-126 impaired suppressive activity of Tregs

**Fig. 4:**
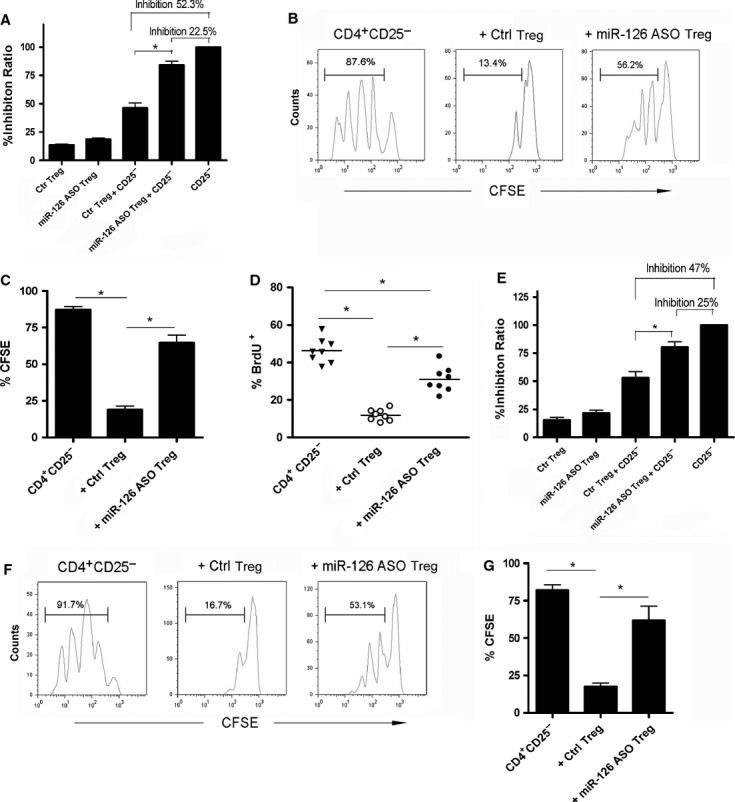
Silencing of miR-126 impaired suppressive activity of Tregs. (**A**) CD4^+^ CD25^−^ T cells (5 × 10^4^) purified from normal Balb/c mice were cultured in the presence of anti-CD3 (2 μg/ml) plus anti-CD28 (4 μg/ml) with or without CD4^+^ CD25^+^ T cells transiently transfected with miR-126 ASO (10 ng) or Scramble control (10 ng) at a ratio of 2:1. Seventy-two hours later, the suppressive capacity of these Tregs was obtained. (**B** and **C**) CD4^+^ CD25^−^ T cells purified from normal Balb/c mice were labelled with CFSE and then cultured in the presence of anti-CD3 (2 μg/ml) and anti-CD28 (4 μg/ml) with or without CD4^+^ CD25^+^ T cells transiently transfected with miR-126 ASO (10 ng) or Scramble control (10 ng) at a ratio of 2:1. Seventy-two hours later, the proliferation of CD4^+^ CD25^−^ T cells was analysed by FACS and calculated. (**D**) CD4^+^ CD25^−^ T cells were purified from normal Balb/c mice and labelled with CFSE. Then, 1 × 10^6^ CFSE-labelled cells were injected into 4T1-bearing nude mice through tail vein with or without CD4^+^ CD25^+^ T cells transfected with miR-126 ASO or Scramble control at a ratio of 2:1. Twenty-four hours later, the 4T1-bearing mice were treated with 2 mg BrdU i.p. every other day up to a cumulative dose of 8 mg BrdU. Then, TILs were collected and the proliferation of CFSE-labelled CD4^+^ T cells were analysed by FACS and calculated. One representative of three independent experiments was shown. CD4^+^ CD25^−^ T cells and CD4^+^ CD25^high^ CD127^−^ T cells (Tregs) were purified from PBMCs of healthy donors (*n* = 6) by FACS sorting. The suppressive activity of Tregs transfected with miR-126 ASO also were determined by [^3^H]-incorporation assay (**E**) and CFSE labelling assay (**F** and **G**) as described above. *P < 0.05.

We next investigated whether miR-126 silencing could influence the suppressive activity of Tregs. As shown in Figure 4A–C, we found that silencing of miR-126 significantly impaired the inhibitory capacity of Tregs on the proliferation of CD4^+^CD25^−^T cells *in vitro* (*P* < 0.05). To confirm this result, we further observed the possible effect of Tregs on the proliferation of CD4^+^CD25^−^ T cells *in vivo* using adoptive cell transfer assay. Expectedly, the proliferation of CFSE-labelled CD4^+^ T cells increased significantly in miR-126 ASO-transfected Tregs cotransferred group compared with that in control group (Fig. 4D, *P* < 0.05). We further confirmed these findings in human Tregs and found that miR-126 ASO also impaired the suppressive activity of human Tregs *in vitro* (Fig. 4E–G, *P* < 0.05). These findings demonstrated that silencing of miR-126 could impair suppressive activity of Tregs.

### MiR-126 regulated Foxp3 expression through PI3K-Akt pathway

**Fig. 5:**
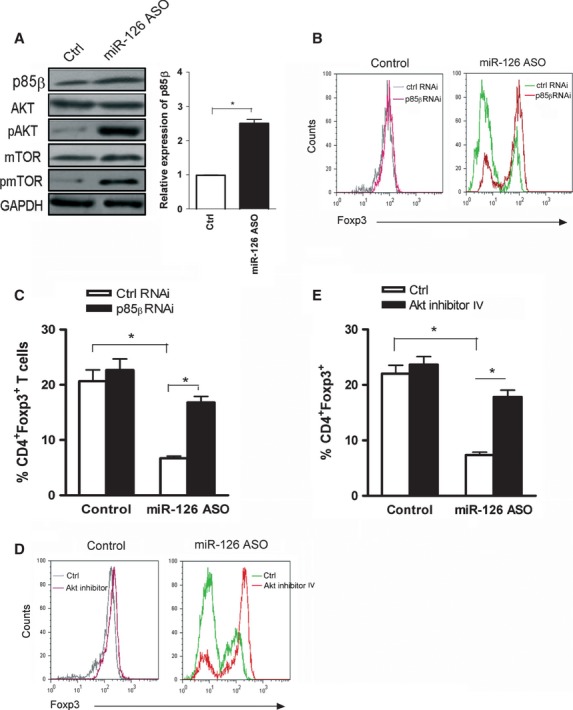
MiR-126 regulated Foxp3 expression through PI3K-Akt pathway. CD4^+^ CD25^+^ T cells (Tregs) purified from splenocytes of normal Balb/c mice by MACS were transiently transfected with miR-126 ASO (10 ng) or Scramble control (10 ng), and then cultured in the presence of anti-CD3/CD28 plus IL-2 (50 IU/ml) for 48 h. The expression of p85β, Akt and mTOR or phosphorylation level of Akt and mTOR were analysed by Western blot (**A**). (**B**) CD4^+^CD25^+^ T cells were transiently transfected with miR-126 ASO (10 ng) or Scramble control plus p85β RNAi (5 ng) or control RNAi (5 ng). Then, cells were cultured in the presence of anti-CD3/CD28 plus IL-2 (50 IU/ml) for 48 h. The expression of Foxp3 was analysed by FACS. (**C**) CD4^+^CD25^−^ T cells purified from normal Balb/c mice by MACS were stimulated with anti-CD3/CD28 antibody in the presence of TGF-β and IL-2 for 24 h, and then were transiently transfected with miR-126 ASO (10 ng) or scramble control (10 ng) plus p85β RNAi (5 ng) or control RNAi (5 ng). After 4 days, the proportion of Foxp3^+^ in CD4^+^ T cells was analysed by FACS and calculated. (**D**) CD4^+^CD25^+^ T cells were transiently transfected with miR-126 ASO (10 ng) or Scramble control. Then, cells were cultured with anti-CD3/CD28 plus IL-2 in the presence of Akt inhibitor IV (1 μM) for 48 h. The expression of Foxp3 was analysed by FACS. (**E**) CD4^+^CD25^−^ T cells purified from normal Balb/c mice by MACS were stimulated with anti-CD3/CD28 antibody in the presence of TGF-β and IL-2 for 24 h, and then were transiently transfected with miR-126 ASO (10 ng) or scramble control (10 ng). These cells were cultured in the presence of Akt inhibitor IV (1 μM) for 4 days, the proportion of Foxp3^+^ in CD4^+^ T cells was analysed by FACS and calculated. One representative of three independent experiments was shown. *P < 0.05.

Recent evidence showed that PI3K/Akt pathway was critical for the expression of Foxp3 in Tregs [10, 11]. And other research works reported miR-126 could target to p85β and regulate the transduction of PI3K/Akt pathway [17, 18]. Then, the direct effect of miR-126 mimics on the expression of p85β in Tregs was detected and similar result was found that miR-126 mimics could obviously inhibit p85 expression in Tregs (data not shown). Next, we further investigated whether miR-126 silencing could enhance the expression of p85β and successively regulate the transduction of TCR-mediated PI3K/Akt pathway which ultimately influenced the expression of Foxp3 in Tregs. As shown in Figure 5A, the expression of p85β in miR-126 ASO-transfected group increased about 2.5 times (*P* < 0.05). Moreover, the level of phosphorylation of Akt and phosphorylation of mTOR increased significantly in miR-126 ASO-transfected group compared with those in control group (Fig. 5A), indicating that miR-126 could regulate the activation of PI3K/Akt pathway in Tregs. Consistently, we also found that miR-126 ASO significantly enhance the expression of p85β, as well as phosphorylated Akt and phosphorylated mTOR, in the induction of Tregs (Supporting Information Figure S2, *P* < 0.05). To further confirm whether this enhanced activation of PI3K-Akt pathway was responsible for reduced expression of Foxp3 in Tregs, we further cotransfected p85β RNAi into Tregs and found that p85β RNAi could abrogate the effect of miR-126 ASO on the expression of Foxp3 in Tregs (Fig. 5B, *P* < 0.05). Furthermore, p85β RNAi also could reverse the effect of miR-126 ASO on Tregs induction (Fig. 5C, *P* < 0.05). To investigate the efficiency and specificity of p85β RNAi on Tregs in miR-126 ASO-transfected group, we further detected the expression of total p85, p85α and p85β on Tregs in miR-126 ASO-transfected group. Our data showed that p85β RNAi could significantly reduce the expression of p85β in miR-126 ASO-transfected Tregs (Supporting Information Figure S3A and B, *P* < 0.05). However, p85β RNAi did not alter the expression of p85α in miR-126 ASO-transfected Tregs (Supporting Information Figure S3B, *P* > 0.05). Finally, to confirm this finding, Akt inhibitor IV also was used and similar results were obtained (Fig. 5D and E, *P* < 0.05). These data strongly suggested that silencing of miR-126 reduced the expression of Foxp3 through up-regulating PI3K/Akt pathway, which ultimately altered the induction and suppressive function of Tregs.

### Silencing of miR-126 in Tregs endowed effective antitumour effect of CD8^+^ T cells in a murine mammary cancer model

**Fig. 6:**
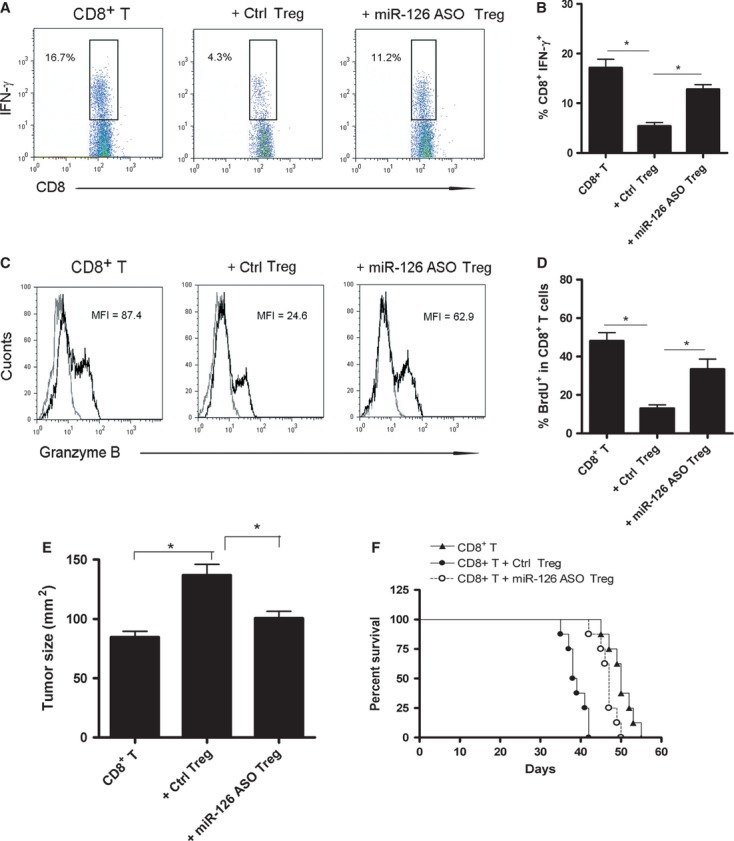
Silencing of miR-126 in Tregs endowed effective antitumour effect of CD8^+^ T cells *in vivo*. Balb/c mice were immunized with inactivated 4T1 tumour cells three times, with 1-week interval. Then CD8^+^ T cells were sorted from splenocytes and re-stimulated with inactivated 4T1 tumour cells for 24 h *in vitro* and then transferred with or without miR-126 ASO-transfected Tregs at a ratio of 2:1 into syngeneic 4T1-bearing nude mice (*n* = 8). Ten days later, TILs were obtained. The percentage of IFN-γ secreting CD8^+^ T cells (**A** and **B**) or the expression of Granzyme B on CD8^+^T cells (**C**) was analysed by FACS respectively. (**D**) The proliferation of CD8^+^ T cells were obtained by BrdU incorporation assay and calculated. (**E**) The tumour size in each group was obtained. (**F**) The survival time of each group was also observed. One representative of three independent experiments was shown. *P < 0.05.

Given that silencing of miR-126 could impair the suppressive activity of Tregs, we next explored the possible application of miR-126 ASO on the function of Tregs *in vivo* using an adoptive cell transfer assay in a murine mammary cancer model. As shown in Figure 6A and B, IFN-γ production in CD8^+^T cells in control ASO-transfected Tregs cotransferred group was 4.72%, while it increased dramatically to 12.06% in miR-126 ASO-transfected Tregs cotransferred group (*P* < 0.05). Furthermore, we found that the expression of Granzyme B and the proliferation of CD8^+^T cells in miR-126 ASO-transfected Tregs cotransferred group also significantly increased compared with those in control group (Fig. 6C and D, *P* < 0.05). Consistently, the size of the tumour mass was significantly elevated in control ASO-transfected Tregs cotransferred group compared with that in CD8^+^T cells transferred group, while it decreased obviously in miR-126 ASO-transfected Tregs cotransferred group (Fig. 6E, *P* < 0.05). Finally, the survival time was also significantly prolonged in miR-126 ASO-transfected Tregs cotransferred group compared with that in control group (Fig. 6F, *P* < 0.05). All these data demonstrated that silencing of miR-126 in Tregs could endow effective antitumour effect of CD8^+^ T cells *in vivo*.

## Discussion

MiRNAs are (21–23 nt) endogenous, non-coding RNA molecules that regulate the expression of protein-coding genes through binding to 3′-UTRs of target mRNAs and inducing translational repression of the target gene or degradation of target mRNA [23]. More than 700 miRNAs have been identified in mammals and have been implicated in a wide range of biological functions. Recent evidence showed that miRNAs played a critical role in the development of Tregs [24, 25]. Different from conventional CD4^+^CD25^−^T cells, Tregs expressed unique miRNAs signature [26]. Given the critical role of Tregs in immune response, the investigation involved in regulation of induction and function sustainment of Tregs is valuable. However, up to now, few miRNAs were reported to regulate Tregs induction and suppressive function. It was reported that miR-155 could modify the induction and suppressive function of Tregs through targeting SOCS1 [27, 28]. And, miR-146a was reported to regulate the suppressive function of Tregs through STAT1 [29]. Most recent evidence showed that miR-17-92 cluster could regulate the induction of Tregs [30]. In this study, we first found that silencing miR-126 could alter both the induction and suppressive function of murine and human Tregs. Together with highly expressed miR-126 in Tregs, our data provided evidence that miR-126 acted as an important regulator for adaptive immune response through impacting on the induction and function of Tregs. Similarly, recent evidence showed that miR-126 could regulate the function of CD4^+^Th2 cells and successively alter the development of allergic airways disease [21].

Accumulating data suggested that limited activation of PI3K/Akt pathway was necessary for Tregs development and function. And T-cell receptor signalling controlled Foxp3 expression *via* PI3K, Akt and Mtor [9]. Conversely, constitutive Akt activity could abrogate the induction of Foxp3 in CD4^+^ T cells and reduce suppressive function of Tregs [13]. Previous study showed that miR-126 could repress the expression of p85β and subsequently reduce the activation of PI3K-Akt pathway, regulating ischaemic angiogenesis and tumour growth [17, 18]. Consistently, our data showed that silencing of miR-126 could enhance the activation of PI3K-Akt pathway in Tregs through up-regulation expression of p85β. Furthermore, p85β RNAi could reverse the effect of miR-126 silencing on Tregs induction and suppressive function. And this finding also was confirmed by the use of Akt inhibitor, combining these data suggested that miR-126 regulated the induction and function of Tregs through targeting p85β and subsequently altering PI3K-Akt pathway activity. Recently, one group reported that overexpression of miR-126 could cause T-cell hyperactivity through targeting to DNA methyltransferase 1 (DNMT1) [20]. And, other group reported that in the absence of DNMT1, TCR signalling was sufficient for induction of Foxp3 expression in CD4^+^T cells [31]. Thus, it seemed that other potent target molecules of miR-126, which were not screened and investigated in this study, might have also contributed to the effect of miR-126 on the induction and function sustainment of Tregs, which still remains to be explored in successive study.

Silencing of specific miRNA, such as using antisense oligonucleotides (ASO), has been developed to be an important strategy for uncovering miRNA biology and emerged as a ultimately novel means to the treatment of various disease [32–34]. As such, silencing microRNA-155 could ameliorate the development of experimental autoimmune encephalomyelitis [35]. And silencing of miR-15 could protect against cardiac ischaemic injury [36]. Our previous study showed that depletion of Tregs could enhance the antitumour function of TILs in adoptive cell transfer assay [37], which was consistent with others' reports [38, 39], suggesting that immunotherapy targeted to Tregs might be an a useful strategic alternative for ACT immunotherapy to enhance antitumour efficacy. Here, we further showed that silencing of miR-126 could significantly impair the suppressive effect of Tregs and finally endow effective antitumour effects of CD8^+^ T cells *in vivo*. This finding suggested that miR-126 might be a novel potential target in Tregs-based therapeutic strategy for immunotherapy against tumour.

Altogether, our present study showed that miR-126 played an important role in the induction and function sustainment of Tregs through regulating PI3K/Akt pathway. We further demonstrated that silencing of miR-126 on Tregs could endow an effective antitumour effect of CD8^+^ T cells *in vivo*. These data provided a novel insight on the functional role of miR-126 in Tregs and might help for the development of new therapeutic strategy based on Tregs for promoting T-cell immunity–related disease.

## Supporting information

**Figure S1** Silencing of miR-126 impaired the expression of Foxp3 on Tregs.

**Figure S2** Silencing of miR-126 enhanced the expression of p85beta, pAkt and pmTOR in the induction of Tregs.

**Figure S3** p85beta RNAi downregulated the expression of p85beta in miR-126 ASO transfected Tregs.
