# Supplementary figures and images for: MicroRNA-126 regulates the induction and function of CD4+ Foxp3+ regulatory T cells through PI3K/AKT pathway
Source: J Cell Mol Med. 2013 Jan 10;17(2):252–64. doi: 10.1111/jcmm.12003 (PMC3822588; doi:10.1111/jcmm.12003)

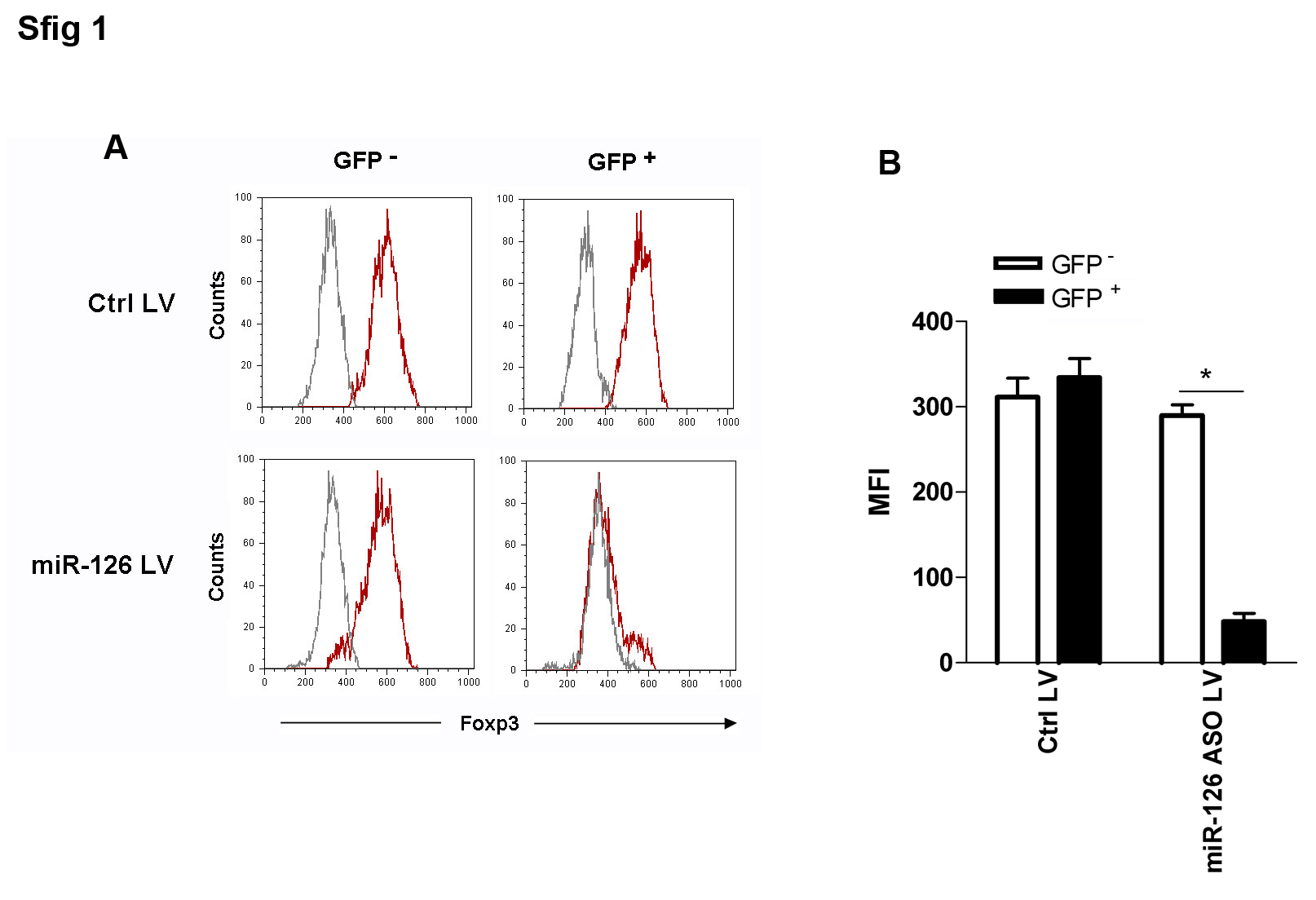

Supplement: Supplementary file 1 [file jcmm0017-0252-SD1.tif]

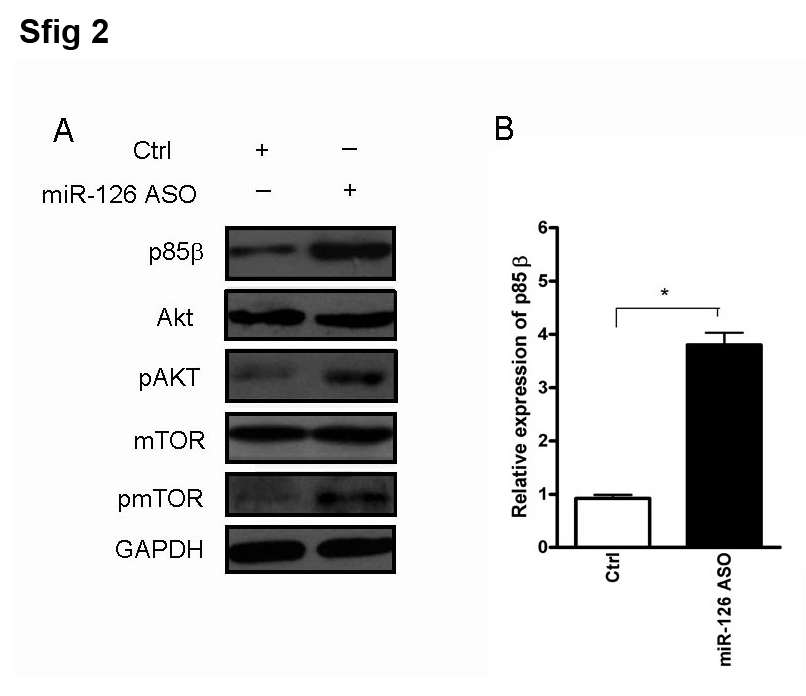

Supplement: Supplementary file 2 [file jcmm0017-0252-SD2.tif]

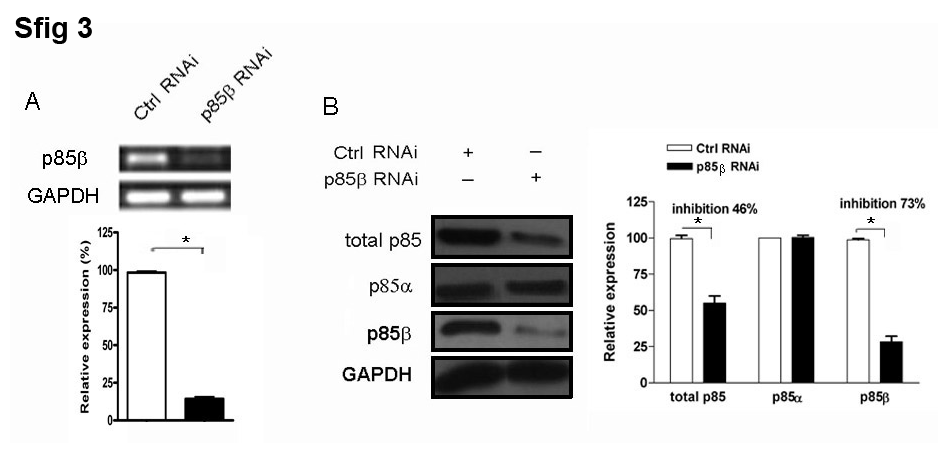

Supplement: Supplementary file 3 [file jcmm0017-0252-SD3.tif]
